# Dark Triad and Resilience to Personal Loss: Evidence from the 2023 Türkiye-Syria Earthquakes

**DOI:** 10.1177/00302228251326147

**Published:** 2025-03-12

**Authors:** Hatime Kamilçelebi, Martijn J. Burger

**Affiliations:** 1 Faculty of Applied Science, 187469 Kırklareli University, Kırklareli, Türkiye; Faculty of Management, Open University of the Netherlands, Heerlen, The Netherlands; 2 Erasmus Happiness Economics Research Organization, 6984 Erasmus University Rotterdam, Rotterdam, The Netherlands; Faculty of Economics, University of Johannesburg, Johannesburg, South Africa; Faculty of Management, Open University of the Netherlands, Heerlen, the Netherlands

**Keywords:** loss, subjective well-being, resilience, Türkiye, dark triad

## Abstract

Can narcissism help foster resiliency against adversity? In this study, we used Turkish panel data, to examine whether narcissism can buffer the negative impact of personal loss on change in subjective well-being in the wake of the Türkiye-Syria earthquakes of 2023. Results show that the adaptation to personal loss was stronger for individuals high on narcissism. At the same time, we found no evidence that individuals high on Machiavellianism or psychopathy – other Dark Triad traits - were better able to adapt to personal loss.

## Introduction

Over the past years, an increasing number of studies have examined the subjective well-being effects of adverse events, including 9/11 (Fredrickson et al., 2003), Boston marathon bombing (Clark et al., 2020), the Paris terrorist attacks (Coupe, 2017), the Greek bailout referendum (Arampatzi et al., 2020), and the Fukushima disaster (Rehdanz et al., 2015). Where it has generally been found that adverse events have a negative impact on subjective well-being, some people appear to be more resilient to adverse events than others. In this regard, positive traits such as emotional intelligence (Armstrong et al., 2011), optimism (Fredrickson et al., 2003), and interpersonal trust (Jovanović et al., 2023) have been found to buffer against adversity.

At the same time, there has been limited attention to the question whether *negative personality traits* buffer against adversity. Most notably, the traits of the Dark Triad (narcissism, Machiavellianism. and psychopathy) have been predominantly associated with negative behaviors, such as fraud (Rijsenbilt & Commandeur, 2013), theft (Lyons & Jonason, 2015), bullying (Davis et al., 2022), and animal cruelty (Kavanagh et al., 2013). Following Paulhus and Williams (2002) narcissism is associated with a magnificent self-view, Machiavellianism is related to interpersonal manipulation and skeptical view of others, and psychopathy is characterized by antisocial impulsivity and heartlessness. Although the Dark Triad personality traits have all their unique features, they share a cold interpersonal orientation, and display of selfish behavior (Lyons et al., 2019). Despite this would suggest that individuals high on narcissism, Machiavellianism, and psychopathy would react and adapt rather similar to adversity, recent studies have pointed out that there are notable differences in coping behavior between the three dark traits.

As pointed out by Lyons et al. (2019) and Szabó, Kun, and Balogh et al. (2022), there are several reasons to believe that particularly (grandiose) narcissism buffers against adverse events. Narcissism has been associated with increased mental toughness or the ability to cope under pressure (Vaughan et al., 2018) as well as reduced emotional reactivity to stress (Papageorgiou et al., 2019). Hyun and Ku (2021) argue that narcissists have a pro-active coping style, characterized by viewing anticipated stressors as opportunities and goals rather than as threats, leading to effective stress handling. Indeed, narcissists seem to exhibit a greater flexibility in dealing with stress, which positively affects their mental health (Ng et al., 2014). In this regard, Zhu et al. (2023) have also pointed at a positive relationship between narcissism and psychological capital, while Onley et al. (2013) have found a positive relationship between narcissism and mental toughness.

Where might this relationship between narcissism and mental toughness originate from? The mourning process represents an adjustment to a significant loss. To navigate this process, individuals require positive internalizations and supportive external relationships. In contrast, narcissistic individuals may struggle with the mourning process. Instead of experiencing grief, they often feel a profound sense of abandonment, accompanied by separation anxiety and episodes of narcissistic rage (Hägglund, 1975). To manage these overwhelming emotions, they tend to cling to grandiose fantasies – avoidance behaviors to escape grief (Durvasula, 2015) - and maintain a high-level of self-esteem and confidence, which can serve as a psychological buffer against distress. Their focus shifts to seeking new relationships and sources of gratification, aiming to restore their sense of being needed and valued (Hägglund, 1975), herewith stimulating mental toughness.

The relationships between other Dark Triad traits and resilience to adverse events are less clear. Studies by Onley et al. (2013) and Bagheri Sheykhangafshe et al. (2021) report a negative association between psychopathy and mental toughness. Individuals high on psychopathy tend to cope less well in the wake of adversity. Despite emotional detachment associated with psychopathy, their difficulty with forming meaningful relationships can result in a lack of social support. Furthermore, individuals high on psychopathy lack self-control (Furnham et al., 2013) and tend to act more impulsive and irresponsible (Onley et al., 2013), which can result in maladaptive coping mechanisms such as substance abuse. Finally, individuals high on psychopathy tend to score lower on emotional intelligence (Miao et al., 2019) and lack a positive mood which may hinder coping (Lyons et al., 2019).

The absence of a buffering effect for people high on Machiavellianism is less well-documented. Some scholars find that Machiavellianism could lead to worse well-being outcomes after stress due to lower emotional intelligence and lower positive mood (Miao et al., 2019), which is possibly associated with high levels of cynicism. Others report that Machiavellians are like narcissists strongly goal-driven (Szabó et al., 2022) and by prioritizing their own goals they tend to navigate adversity. However, exploiting situations for personal gains may harm social relationships in the longer run. More elaborate discussions on the relationship between the Dark Triad traits and coping can be found in the work of Lyons et al. (2019) and Szabó, Kun, and Balogh et al. (2022).

In this study, we build on previous event studies by using panel data in the wake of an adverse event, the 2023 Türkiye-Syria Earthquakes. On February 6, an earthquake struck central Türkiye and the north-west Syrian Arab Republic. Over 50,000 people died in Türkiye, while 1.5 million people were left homeless. Specifically, we examine adaptation to personal loss (in terms subjective well-being) in the months after the earthquake and gauge whether narcissism, Machiavellianism, and psychopathy moderate the relationship between personal loss and change in subjective well-being.

The remainder of this paper is organized as follows. The data and methodology are introduced in *Data and Methodology*. Descriptive statistics and an empirical analysis are provided in *Results*, while *Discussion* concludes.

## Data and Methodology

### Participants and Procedure

In this research, we utilized data obtained from two surveys conducted in Türkiye following the earthquakes that occurred in February 2023. The initial survey took place from the final week of March to the second week of May 2023 (T1), while the follow-up survey was held from the second week of September to the third week of October 2023 (T2). Both surveys were administered online throughout Türkiye using convenience sampling via a provided link. Participation in the survey was voluntary, and respondents did not receive compensation for their involvement. The survey was distributed through Google Forms, and the average time taken to complete it was 20 minutes. All 1100 individuals who completed the first wave of the survey were invited to partake in the second wave, resulting in a sample of 608 individuals who completed both surveys. The data collection procedure was reviewed and approved by the Scientific Research and Publication Ethics Committee of the Rectorate of Kirklareli University (reference no: E-35523585-199).^1^ The respondents provided their written informed consent to participate in this study. The average age of respondents was 39 years, and the majority of respondents was male (65%) and had a university degree (89%).^2^

### Variables

#### Personal Loss During Earthquake

The personal loss variable is a Boolean dummy variable that takes the value of one if the respondent indicated (1) he or she survived but was injured and/or (2) his or her house was damaged or destroyed and/or (3) one or more family members were injured or passed away and/or the family’s house was damaged or destroyed. This variable was measured during the first wave of the study.

#### Change in Subjective Well-Being

To measure resilience to adverse events, we look at the change in life satisfaction score, which can be considered an evaluative measure of subjective well-being and is commonly used in the well-being literature. Specifically, respondents had to answer the question: ‘Taking all things together, how satisfied are you with your life these days? 0 = completely dissatisfied, 10 = completely satisfied’. This single-item measure is used frequently in the study of subjective well-being (Diener et al., 2013) and perform relatively well as compared to multi-item scales like Diener’s Satisfaction with Life Scale, albeit with lower reliabilities (Schimmack & Oishi, 2005). This variable was part of the questionnaire in both survey waves and, hence, were are able to examine change in subjective well-being over time, which is used to gauge to what extent people adapt to adverse events.

#### Narcissism and Other Dark Triad Traits

We use the Turkish translation (Özsoy & Ardiç, 2017) of the Dirty Dozen scale (Jonason & Webster, 2010) to measure the Dark Triad traits. The dirty dozen scale encompasses a 9-point Likert-type scale (1 = strongly disagree; 9 = strongly agree) and 4 items for each trait. Examples of statements include ‘I tend to want others to admire me’ (narcissism), ‘I tend to manipulate others to get my way’ (Machiavellianism), and ‘I tend to be callous or insensitive (psychopathy). “Better”). The Dark Triad personality traits were only measured in the first wave. Cronbach’s alpha for the narcissism index (0.87), Machiavellianism index (0.85), and psychopathy index (0.73) indicated that the respective indices are internally consistent.

#### Control Variables

We control for gender, age, and education level as these factors have been previously associated with Dark Triad traits (see e.g. Szabó et al., 2022). Table 1 includes descriptive statistics for all variables included in the study, while Table 2 provides the correlation matrix for the main variables of interest.

**Table 1.** Descriptive Statistics (*N* = 608).

	Mean	Standard deviation	Minimum	Maximum	Skewness	Kurtosis
Continuous variables						
Change in life satisfaction (T2-T1)	0.41	2.35	−10	10	0.1	5.0
Narcissism (T1)	4.96	2.10	1	9	−0.1	2.0
Machiavellianism (T1)	2.17	1.80	1	9	1.8	6.3
Psychopathy (T1)	2.29	1.44	1	9	1.5	5.4
Age	38.82	9.19	1	9	0.6	3.1
Categorical variables	%					
Personal loss during earthquake	9.4					
Female	34.5					
University degree	88.9

**Table 2.** Correlation Matrix (*N* = 608).

	(1)	(2)	(3)	(4)	(5)
(1) Change in life satisfaction (T2-T1)	1.00				
(2) Personal loss during earthquake	0.03	1.00			
(3) Narcissism (T1)	−0.02	0.02	1.00		
(4) Machiavellianism (T1)	−0.00	0.01	0.33	1.00	
(5) Psychopathy (T1)	0.03	0.02	0.15	0.53	1.00

### Self-Selection and Attrition Bias

Given the longitudinal design, a potential caveat is that our data might suffer from self-selection selection bias and panel attrition bias. Attrition bias arises if respondents drop out for the panel non-randomly, namely, when attrition is interrelated with variables of interest. We examine attrition bias using the demographic data, Dark Triad scores, and subjective well-being scores of the respondents that were obtained in the first questionnaire. Overall, our attrition is 44.7% which is not uncommon in long-term panel studies in which attrition rates typically vary between 30% and 70% (e.g., Gustavson et al., 2012).

Using logistic regression, we compared the respondents who completed both questionnaires and respondents who only filled out one questionnaire. The results are presented in Table 3. The scores on the main variables of interest between individuals who participated in both waves and those who only participated in one wave did not significantly differ. Hence, we conclude that the selection effect regarding our main variables of interest is limited. However, respondents who had a university degree, were more likely to complete both waves of the survey.

**Table 3.** Self-Selection in Sample—Participation in Two Rounds Versus Only First Round – Odds Ratio’s.

Dependent variable: Participation in two waves (0-1)	(1) Odds ratio (95%CI)
Life satisfaction (T1)	1.04 (0.99–1.09)
Personal loss during earthquake (T1)	0.96 (0.63–1.47)
Narcissism (T1)	1.04 (0.98–1.11)
Machiavellianism (T1)	0.95 (0.85–1.05)
Psychopathy (T1)	1.00 (0.91–1.11)
Female	0.95 (0.72–1.25)
Age	1.00 (0.99–1.01)
University degree	3.11** (2.36–4.09)
Observations	1100

**p < .01. Confidence intervals estimated using robust standard errors.

## Results

### Main Findings

To examine whether Dark Triad traits moderate the relationship between personal loss during the earthquake and change in subjective well-being in the months after the earthquake, we utilize ordinary least squares (OLS) regressions. Table 4 provides the main findings. Overall, we find that people that experienced personal loss in the family during the earthquake score lower on life satisfaction in both T1 and T2, but that the difference between people that experienced personal loss and did not experience personal loss is becoming smaller over time. Please note that between T1 and T2 the life satisfaction of both people that experienced personal loss and people that did not experience personal loss improved. This can be explained by the fact that also the subjective well-being of people that did not experience loss was negatively affected by the earthquakes.

**Table 4.** Main Results – Linear Regression.

Dependent variable: Change in life satisfaction (T2-T1)	(1)	(2)	(3)	(4)
Personal loss during earthquake (T1)	0.21 (0.34)	0.27 (0.37)	0.29 (0.37)	0.22 (0.35)
Narcissism (T1)	−0.10 (0.06)#	−0.04 (0.05)	−0.04 (0.05)	−0.10 (0.06)#
Machiavellianism (T1)	0.01 (0.07)	−0.00 (0.07)	0.01 (0.07)	0.02 (0.07)
Psychopathy (T1)	−0.04 (0.08)	−0.06 (0.08)	−0.06 (0.08)	−0.04 (0.08)
Personal loss during earthquake * narcissism (T1)	0.57 (0.15)**			0.60 (0.17)**
Personal loss during earthquake * Machiavellianism (T1)		0.16 (0.24)		−0.14 (0.26)
Personal loss during earthquake * psychopathy (T1)			−0.09 (0.26)	0.01 (0.21)
Female	−0.12 (0.21)	−0.18 (0.22)	−0.17 (0.21)	−0.12 (0.21)
Age	−0.02 (0.01)	−0.02 (0.01)#	−0.02 (0.01)	−0.02 (0.01)
University degree	0.84 (0.34)*	0.86 (0.35)*	0.83 (0.34)*	0.82 (0.35)*
Observations	608	608	608	608

Robust standard errors in parentheses. **p < .01; *p < .05; #*p* < .10. Narcissism (T1), Machiavellianism (T1), and Psychopathy (T1) are all mean-centered.

At the same time, this adaptation effect is insignificant (Column 1: b = 0.28, *p* = .34), signifying that there is considerable heterogeneity in the adaptation effect. Moving to the buffering effects of the Dark Triad traits, we find that, in line with our hypothesis and previous work, narcissism moderates the relationship between experience personal loss and change in life satisfaction (Column 2: b = 0.57, se = 0.15, *p* < .01; see also Figure 1). Although narcissism is weakly negatively associated with change in life satisfaction (b = -0.10, *p* = .084), the life satisfaction gap between people that are high on narcissism and experienced personal loss and people that did not experience personal loss decreased. Concurrently, the life satisfaction gap between people that are low on narcissism and experienced personal loss and people that did not experience personal loss increased. We found that the moderating effects of Machiavellianism and psychopathy were insignificant.

**Figure 1.**
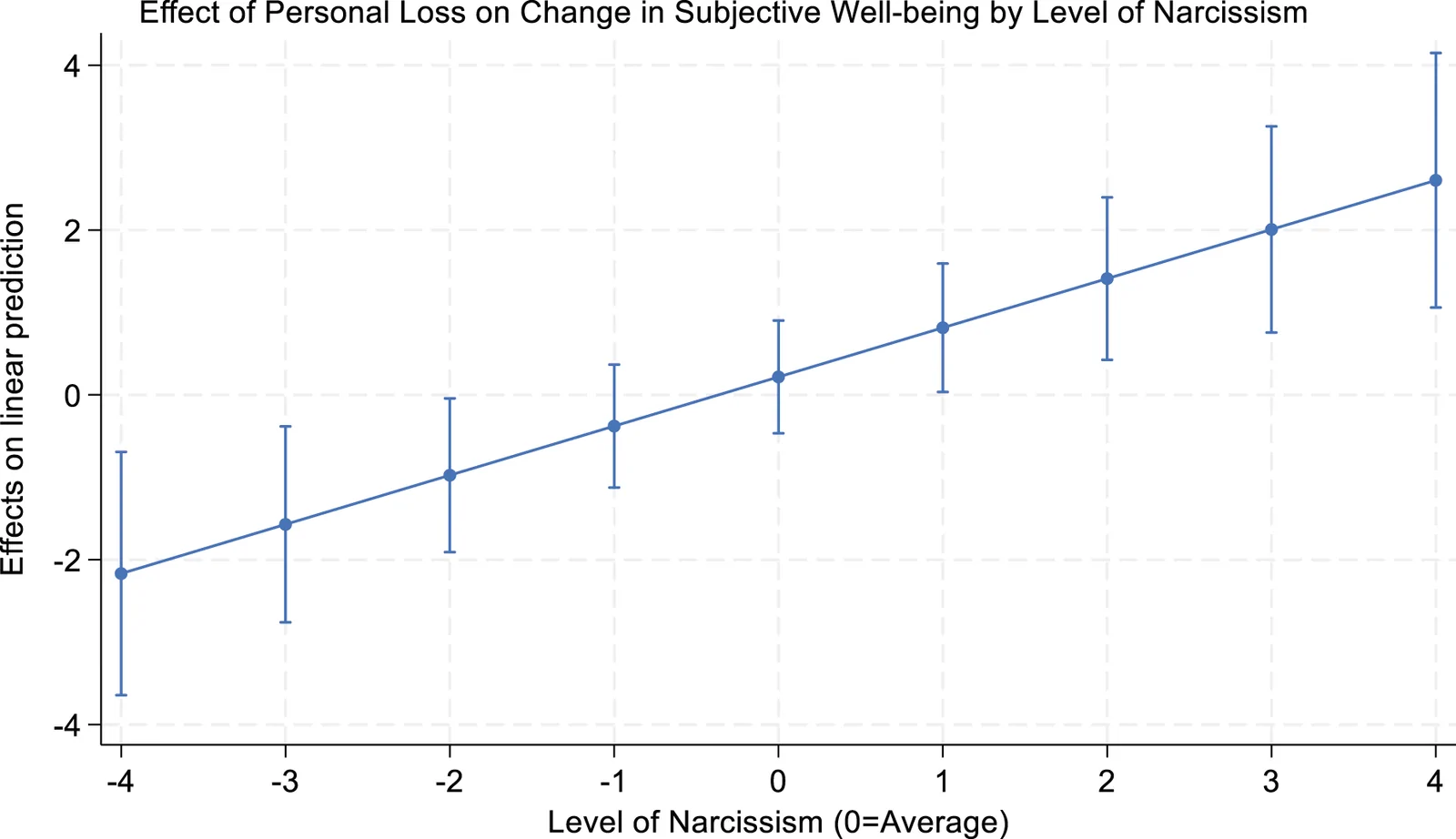
The moderating effect of narcissism.

### Robustness Analyses

We ran several robustness analyses, where we examined whether our results are sensitive to variable definition and inclusion of covariates. First, we re-estimated Column 4 of Table 4 using 2 different definitions of being affected by the 2023 earthquakes. These results are presented in Table 5. First, we used the original definition, but now also included the injury, death and/or destruction of houses of friends or acquaintances in our definition of being affected by the earthquake (Table 5, Column 1). Second, we defined being affected by the earthquake by being present in the earthquake area at the time of the earthquake (Table 5, Column 2). When we apply different definitions of adversity, our main results do not change.

**Table 5.** Robustness Analysis – Alternative Definitions of Personal Loss.

Dependent variable: Change in life satisfaction (T2-T1)	(1)	(2)
Personal loss during earthquake and/or loss of friends/acquaintances	0.18 (0.21)	
Present in earthquake area		0.21 (0.35)
Narcissism (T1)	−0.09 (0.06)	−0.07 (0.06)
Machiavellianism (T1)	−0.03 (0.08)	0.02 (0.08)
Psychopathy (T1)	−0.10 (0.09)	−0.06 (0.08)
Personal loss during earthquake and/or loss of friends/acquaintances * narcissism (T1)	0.21 (0.10)#	
Personal loss during earthquake and/or loss of friends/acquaintances * Machiavellianism (T1)	0.16 (0.18)	
Personal loss during earthquake and/or loss of friends/acquaintances * psychopathy (T1)	0.15 (0.17)	
Present in earthquake area * narcissism (T1)		0.35 (0.17)*
Present in earthquake area * machiavellianism (T1)		−0.05 (0.29)
Present in earthquake area * psychopathy (T1)		0.14 (0.29)
Covariates	Yes	Yes
Observations	608	608

Robust standard errors in parentheses. **p < .01; *p < .05; #*p* < .10.

Narcissism (T1), Machiavellianism (T1), and Psychopathy (T1) are all mean-centered.

In addition, we conducted additional regressions including additional covariates that potentially confound the relationships between personal loss, dark-triad and change in life satisfaction. In particular, we ran regressions (Table 6) where we controlled for:

(1) The life satisfaction level in wave 1. Results are presented in Table 6, Column 1 Although we might have expected that people higher on subjective well-being would be more resilient, the limited dependent nature of the life satisfaction response scale (0-10) could contribute to the finding that potential gains in life satisfaction are greater for less happy people (see also Arampatzi et al., 2020). Our main results regarding the moderation effect of narcissism hold.
(2) Other personality traits. We controlled whether our findings regarding the Dark Triad traits is not driven by other personality characteristics. Including the brief version of the Big Five (Table 6, Column 2) using the scale of Gosling et al. (2003) that has been translated by Atak (2013) in Turkish, our main results hold.

**Table 6.** Robustness Analysis – Additional Covariates.

Dependent variable: Change in life satisfaction (T2-T1)	(1)	(2)
Personal loss during earthquake	−0.07 (0.27)	0.16 (0.34)
Narcissism (T1)	−0.04 (0.05)	−0.11 (0.06)#
Machiavellianism (T1)	−0.01 (0.07)	−0.01 (0.08)
Psychopathy (T1)	−0.05 (0.07)	−0.04 (0.08)
Personal loss during earthquake * narcissism (T1)	0.31 (0.13)**	0.62 (0.17)**
Personal loss during earthquake * Machiavellianism (T1)	0.12 (0.20)	−0.16 (0.25)
Personal loss during earthquake * psychopathy (T1)	0.11 (0.19)	−0.01 (0.21)
Life satisfaction (T1)	−0.47 (0.04)**	
Female	0.04 (0.18)	−0.07 (0.22)
Age	−0.00 (0.01)	−0.01 (0.01)
University degree	1.18 (0.30)**	0.83 (0.34)*
Big five personality traits	No	Yes
Observations	608	608

Robust standard errors in parentheses. **p < .01; *p < .05; #*p* < .10.

Narcissism (T1), Machiavellianism (T1), and Psychopathy (T1) are all mean-centered.

## Discussion

The present study examined the buffering effects of Dark Triad traits to adverse events, in a sample of Turkish respondents in the wake of the 2023 Türkiye-Syria Earthquake. We found that respondents high on narcissism that experienced personal loss adapted better to adversity. Our findings are in line with the literature on Dark Triad traits and resilience, which has found that narcissism may serve as a buffer against adverse events due to its association with increased mental toughness, reduced emotional reactivity to stress, and a proactive coping style that frames stressors as opportunities rather than threats (Hyun & Ku, 2021; Lyons et al., 2019; Szabó et al., 2022). We did not find a moderating effect of Machiavellianism and psychopathy, where the previous literature has found mixed effects for the role of Machiavellianism in the adaptation process and a negative buffering effect of psychopathy. Explanations for these findings are that psychopathy and Machiavellianism are negatively associated with resilience and mental toughness, likely due to traits like impulsivity, irresponsibility, low emotional intelligence, and a lack of positive mood (Furnham et al., 2013; Miao et al., 2019; Onley et al., 2013).

Our findings support the adaptive potential of narcissism, as proposed by the stress-buffering hypothesis (Szabó et al., 2022) and earlier, but also highlight the need for a more nuanced understanding of how these traits interact with environmental factors. At the same time, our findings have important implications for psychological interventions or resilience-building programs in those certain characteristics of (grandiose) narcissism such as mental toughness can be harnessed in therapeutic settings to help individuals cope with personal loss. Although it might be unwarranted to promote narcissistic traits in general, some of these traits such as self-confidence and mental toughness, are adaptive in certain contexts. Evidence for narcissism as coping mechanism has also been found in studies on death anxiety, in which it was found that narcissism moderates the relationship between death anxiety and subjective well-being (Zhao et al., 2022).

A theoretical implication of our findings is that greater attention should be given to the bright side of malevolent qualities as well as that Dark Triad personality traits have their own unique characteristics that should be more prominently featured in future research. Our results echo findings in the wider literature on personality and subjective well-being, in which it has been found that particularly grandiose narcissism has been associated with higher levels of subjective well-being, while Machiavellianism and psychopathy are not or negatively associated with subjective well-being (Blasco-Belled et al., 2024). In this regard, narcissism is considered to be ‘on the lighter side’ of Dark Triad traits (Aghababaei & Błachnio, 2015), where the study by Van Groningen et al. (2021) has even shown that narcissism may moderate the relationship between Machiavellianism and psychopathy and subjective well-being.

There are several limitations of this that should be addressed in future research. First, we utilized a single-item measure of life satisfaction. Although this method has been successfully applied in large-scale research to examine the connection between objective factors, such as income, and life satisfaction (e.g., Jebb et al., 2018), single-item measures are typically less reliable than multi-item scales, which may lead to a reduction in the observed effects (see Diener et al., 2013). However, recent research comparing single-item and multi-item life satisfaction scales across three extensive samples has shown strong alignment and, importantly, no consistent differences in their correlations with outcomes (Cheung & Lucas, 2014).

Second, we were only able to study adaptation in the medium run, as we examined change in subjective well-being in the first half-year after the earthquakes. Future studies should further examine how and under what circumstances the Dark Triad personality traits help to adapt to adversity in the short-run (over a period of weeks or months) and long-run (over a period of years). Longer-run studies could help to examine whether the buffering effects of narcissism are sustained or whether they diminish over time. High-frequency pulse surveys (e.g., Arampatzi et al., 2020), Experience Sampling (e.g., Wijngaards et al., 2019) or Day Reconstruction Method (e.g., Bakker et al., 2020) can be employed to capture short-term effects and fluctuations in subjective well-being and coping strategies. Such study could provide us with a better understanding how Dark Triad traits affect resilience in the wake of a disaster.

Third, the current study is based on self-report data and particularly individuals high on narcissism may overreport their subjective well-being (Zuckerman & O’Loughlin, 2009). This could potentially distort the relationship between narcissism and resilience. Hence, there is still a need for further research about narcissism and resilience not only on self-report data but also on behavioral data and observer reports, which can provide a better understanding of the relation between narcissism and resilience and can easily be expanded to other Dark Triad traits.

Fourth and most important of all, our study is only generalizable to a limited extent since it studies a specific event and draws on a convenience sample that is not representative of the Turkish population (Kamilçelebi & Burger, 2024); university graduates might be a specific group and the earthquake might have affected them somewhat differently compared to the general population. The problem in this paper is analogous to the problem of al large reliance on student samples elsewhere in the psychological literature. At the same time, our sample that mostly consists of university graduates can still be valid in showcasing impact of an earthquake. In addition, there is no prior that personality factors would have a different effect in different parts of the population. Although we suspect that it is not likely that our main findings would change considerably when more representative samples are employed, especially given the magnitude of the found effects, additional research is needed to verify this claim.

On a similar note, more research on how contextual factors play a role should be initiated. First, the study has been conducted in Türkiye that has a collectivist culture and it would be interesting to examine whether our findings would hold in more individualistic cultures, where narcissistic traits might be more prevalent or socially accepted. In addition, it would be interesting to examine to what extent our findings are specific to the context of a natural disaster and whether the buffering effect of narcissism might differ in other types of adversity, such as economic crises or pandemics. For example, could narcissism be less adaptive in situations where social cooperation is essential for survival?

## Conclusions

In this study we examined whether Dark Triad traits can contribute to resilience in the face of adversity. The study utilized Turkish panel data to explore whether narcissism, Machiavellianism and psychopathy can mitigate the negative effects of personal loss on changes in subjective well-being – measured by life satisfaction - following the 2023 Türkiye-Syria earthquakes. The findings indicate that individuals with higher levels of narcissism adapted more effectively to personal loss. However, no evidence was found to suggest that those with high levels of Machiavellianism or psychopathy demonstrated greater adaptability to personal loss.

## Notes

1 The study was not preregistered.

2 Data will be made available in the open Mendelay repository upon publication.

## References

[bibr1-00302228251326147] Aghababaei N. Błachnio A. (2015). Well-being and the dark triad. Personality and Individual Differences, 86(November), 365–368. 10.1016/j.paid.2015.06.043

[bibr2-00302228251326147] Arampatzi E. Burger M. Stavropoulos S. Tay L. (2020). The role of positive expectations for resilience to adverse events: Subjective well-being before, during and after the Greek bailout referendum. Journal of Happiness Studies, 21(3), 965–995. 10.1007/s10902-019-00115-9

[bibr3-00302228251326147] Armstrong A. R. Galligan R. F. Critchley C. R. (2011). Emotional intelligence and psychological resilience to negative life events. Personality and Individual Differences, 51(3), 331–336. 10.1016/j.paid.2011.03.025

[bibr4-00302228251326147] Atak H. (2013). The Turkish adaptation of the ten-item personality inventory. Nöro Psikiyatri Arşivi, 50(4), 312–319. 10.4274/npa.y6128 PMC5363422 28360563

[bibr5-00302228251326147] Bagheri Sheykhangafshe F. Abolghasemi A. Kafi Masouleh S. M. (2021). Predicting Resilience based on Dark Triad personality and psychological wellbeing in athletes students. Journal of Arak University of Medical Sciences, 24(2), 230–245. 10.32598/jams.24.2.6151.1

[bibr6-00302228251326147] Bakker A. Burger M. van Haren P. Oerlemans W. Veenhoven R. (2020). Raise of happiness following raised awareness of how happy one feels: A follow-up of repeated users of the happiness indicator website. International Journal of Applied Positive Psychology, 5(3), 153–187. 10.1007/s41042-020-00032-w

[bibr7-00302228251326147] Blasco-Belled A. Tejada-Gallardo C. Alsinet C. Rogoza R. (2024). The links of subjective and psychological well-being with the dark triad traits: A meta-analysis. Journal of Personality, 92(2), 584–600. 10.1111/jopy.12853 37249010

[bibr8-00302228251326147] Cheung F. Lucas R. E. (2014). Assessing the validity of single-item life satisfaction measures: Results from three large samples. Quality of Life Research, 23(10), 2809–2818. 10.1007/s11136-014-0726-4 PMC4221492 24890827

[bibr9-00302228251326147] Clark A. E. Doyle O. Stancanelli E. (2020). The impact of terrorism on individual well-being: Evidence from the Boston Marathon bombing. The Economic Journal, 130(631), 2065–2104. 10.1093/ej/ueaa053

[bibr10-00302228251326147] Coupe T. (2017). The impact of terrorism on expectations, trust and happiness–the case of the November 13 attacks in Paris, France. Applied Economics Letters, 24(15), 1084–1087. 10.1080/13504851.2016.1254335

[bibr11-00302228251326147] Davis A. C. Farrell A. H. Brittain H. Krygsman A. Arnocky S. Vaillancourt T. (2022). The dark triad and bullying in adolescence: A three-wave random intercept cross-lagged panel analysis. Journal of Research in Personality, 96(February), Article 104178. 10.1016/j.jrp.2021.104178

[bibr12-00302228251326147] Diener E. Inglehart R. Tay L. (2013). Theory and validity of life satisfaction scales. Social Indicators Research, 112(3), 497–527. 10.1007/s11205-012-0076-y

[bibr13-00302228251326147] Durvasula R. (2015). Should I stay or should I go: Surviving a relationship with a narcissist. Post Hill Press.

[bibr14-00302228251326147] Fredrickson B. L. Tugade M. M. Waugh C. E. Larkin G. R. (2003). What good are positive emotions in crisis? A prospective study of resilience and emotions following the terrorist attacks on the United States on september 11th, 2001. Journal of Personality and Social Psychology, 84(2), 365–376. 10.1037//0022-3514.84.2.365 PMC2755263 12585810

[bibr15-00302228251326147] Furnham A. Richards S. C. Paulhus D. L. (2013). The dark triad of personality: A 10-year review. Social and Personality Psychology Compass, 7(3), 199–216. 10.1111/spc3.12018

[bibr16-00302228251326147] Gosling S. D. Rentfrow P. J. Swann W. B. (2003). A very brief measure of the Big-Five personality domains. Journal of Research in Personality, 37(6), 504–528. 10.1016/s0092-6566(03)00046-1

[bibr17-00302228251326147] Gustavson K. von Soest T. Karevold E. Røysamb E. (2012). Attrition and generalizability in longitudinal studies: Findings from a 15-year population-based study and a Monte Carlo simulation study. BMC Public Health, 12, 918–1011. 10.1186/1471-2458-12-918 PMC3503744 23107281

[bibr18-00302228251326147] Hägglund T. B. (1975). Mourning and narcissism. Psychiatria Fennica, Yearbook, 151–156.

[bibr19-00302228251326147] Hyun S. Ku X. (2021). Proactive coping mediates the relationship between the narcissism phenotypes and psychological health. Social Behavior and Personality: International Journal, 49(7), 1–15. 10.2224/sbp.10477

[bibr41-00302228251326147] Jebb A. T. Tay L. Diener E. Oishi S. (2018). Happiness, income satiation and turning points around the world. Nature Human Behaviour, 2(1), 33–38. 10.1038/s41562-017-0277-0 30980059

[bibr20-00302228251326147] Jonason P. K. Webster G. D. (2010). The dirty dozen: A concise measure of the dark triad. Psychological Assessment, 22(2), 420–432. 10.1037/a0019265 20528068

[bibr21-00302228251326147] Jovanović V. Sarracino F. Lazić M. Gavrilov-Jerković V. (2023). Well-being and the pandemic: Trust in people matters more than trust in institutions. Psychological Reports, 126(2), 674–687. 10.1177/00332941211061692 34964386

[bibr42-00302228251326147] Kamilçelebi H. Burger M. J. (2024). Fairness perceptions mediate the relationship between income comparisons and subjective well-being: evidence from Türkiye. Applied Economics Letters, 1–5. 10.1080/13504851.2024.2332584

[bibr22-00302228251326147] Kavanagh P. S. Signal T. D. Taylor N. (2013). The Dark Triad and animal cruelty: Dark personalities, dark attitudes, and dark behaviors. Personality and Individual Differences, 55(6), 666–670. 10.1016/j.paid.2013.05.019

[bibr23-00302228251326147] Lyons M. Evans K. Helle S. (2019). Do “dark” personality features buffer against adversity? The associations between cumulative life stress, the dark triad, and mental distress. Sage Open, 9(1), Article 2158244018822383. 10.1177/2158244018822383

[bibr24-00302228251326147] Lyons M. Jonason P. K. (2015). Dark triad, tramps, and thieves. Journal of Individual Differences, 36(4), 215–220. 10.1027/1614-0001/a000177

[bibr25-00302228251326147] Miao C. Humphrey R. H. Qian S. Pollack J. M. (2019). The relationship between emotional intelligence and the dark triad personality traits: A meta-analytic review. Journal of Research in Personality, 78(February), 189–197. 10.1016/j.jrp.2018.12.004

[bibr26-00302228251326147] Ng H. K. Cheung R. Y. H. Tam K. P. (2014). Unraveling the link between narcissism and psychological health: New evidence from coping flexibility. Personality and Individual Differences, 70(November), 7–10. 10.1016/j.paid.2014.06.006

[bibr27-00302228251326147] Onley M. Veselka L. Schermer J. A. Vernon P. A. (2013). Survival of the scheming: A genetically informed link between the dark triad and mental toughness. Twin Research and Human Genetics, 16(6), 1087–1095. 10.1017/thg.2013.66 24074275

[bibr28-00302228251326147] Özsoy E. Ardiç K. (2017). Examining the effects of the dark triad (narcissism, Machiavellianism and psychopathy) on job satisfaction. Yonetim ve Ekonomi, 24(2), 391–406. 10.18657/yonveek.297733

[bibr29-00302228251326147] Papageorgiou K. A. Gianniou F. M. Wilson P. Moneta G. B. Bilello D. Clough P. J. (2019). The bright side of dark: Exploring the positive effect of narcissism on perceived stress through mental toughness. Personality and Individual Differences, 139(March), 116–124. 10.1016/j.paid.2018.11.004

[bibr30-00302228251326147] Paulhus D. L. Williams K. M. (2002). The dark triad of personality: Narcissism, Machiavellianism, and psychopathy. Journal of Research in Personality, 36(6), 556–563. 10.1016/s0092-6566(02)00505-6

[bibr31-00302228251326147] Rehdanz K. Welsch H. Narita D. Okubo T. (2015). Well-being effects of a major natural disaster: The case of Fukushima. Journal of Economic Behavior & Organization, 116(August), 500–517. 10.1016/j.jebo.2015.05.014

[bibr32-00302228251326147] Rijsenbilt A. Commandeur H. (2013). Narcissus enters the courtroom: CEO narcissism and fraud. Journal of Business Ethics, 117(2), 413–429. 10.1007/s10551-012-1528-7

[bibr33-00302228251326147] Schimmack U. Oishi S. (2005). The influence of chronically and temporarily accessible information on life satisfaction judgments. Journal of Personality and Social Psychology, 89(3), 395–406. 10.1037/0022-3514.89.3.395 16248721

[bibr34-00302228251326147] Szabó Z. P. Kun A. Balogh B. E. Simon E. Csike T. (2022). Dark and strong?! The associations between dark personality traits, mental toughness and resilience in Hungarian student, employee, leader, and military samples. Personality and Individual Differences, 186(Part B, February), Article 111339. 10.1016/j.paid.2021.111339

[bibr35-00302228251326147] Van Groningen A. J. Grawitch M. J. Lavigne K. N. Palmer S. N. (2021). Every cloud has a silver lining: Narcissism's buffering impact on the relationship between the Dark Triad and well-being. Personality and Individual Differences, 171(March), Article 110549. 10.1016/j.paid.2020.110549

[bibr36-00302228251326147] Vaughan R. Carter G. L. Cockroft D. Maggiorini L. (2018). Harder, better, faster, stronger? Mental toughness, the dark triad and physical activity. Personality and Individual Differences, 131(September), 206–211. 10.1016/j.paid.2018.05.002

[bibr37-00302228251326147] Wijngaards I. Hendriks M. Burger M. J. (2019). Steering towards happiness: An experience sampling study on the determinants of happiness of truck drivers. Transportation Research Part A: Policy and Practice, 128(October), 131–148. 10.1016/j.tra.2019.07.017

[bibr38-00302228251326147] Zhao N. Liu B. Wang Y. (2022). Examining the relationship between death anxiety and well-being of frontline medical staff during the COVID-19 pandemic. International Journal of Environmental Research and Public Health, 19(20), Article 13430. 10.3390/ijerph192013430 PMC9603769 36294046

[bibr39-00302228251326147] Zhu X. Geng Y. Pan Y. Shi L. (2023). Conspicuous consumption in Chinese young adults: The role of dark tetrad and gender. Current Psychology, 42(23), 19840–19852. 10.1007/s12144-022-03129-4

[bibr40-00302228251326147] Zuckerman M. O’Loughlin R. E. (2009). Narcissism and well-being: A longitudinal perspective. European Journal of Social Psychology, 39(6), 957–972.

